# Vitamin C Deficiency Presenting as an Acute Limp in Childhood

**DOI:** 10.7759/cureus.9182

**Published:** 2020-07-14

**Authors:** Andrew Kyprios

**Affiliations:** 1 Paediatrics, Gloucester Royal Hospital, Gloucester, GBR

**Keywords:** scurvy, vitamin c, limp, pediatrics, irritable hip, transient synovitis, dental decay

## Abstract

A previously well three-year-old boy attended with right knee pain and an antalgic gait. There was no history of fever and bloods including inflammatory markers were normal. A diagnosis of transient synovitis (irritable hip) was made and managed conservatively. One month later, he represented with increasing pain, now localising to his left hip, waking him at night with difficulty weight-bearing. No effusion was seen on ultrasound and bloods remained normal. He was discharged home but came back a week later with worsening polyarticular pain, a new maculopapular rash, significant bruising and new dental decay. A clinical diagnosis of vitamin C deficiency was made secondary to dietary insufficiency, and this was confirmed on blood testing. Within six weeks, his symptoms had fully improved on oral ascorbic acid (vitamin C) and he was engaging in dietetic input and reward systems to maintain a more balanced diet.

## Introduction

Vitamin C is essential to maintain the normal structure and function of teeth, bones, connective tissues and many other biochemical pathways including immune function [1]. It has been known for a long time that dietary sources such as citrus fruit, green vegetables and tomatoes are key to maintaining many of these processes, and as such when a child is deficient, symptoms may manifest across many different diagnostic lines, including haematological, rheumatological, inflammatory and infection [2]. Deficiency may be secondary to reduced intake, reduced absorption or in some cases a metabolic condition that prevents in vivo conversion to a functional form. In adults, the causes of relative deficiency are most commonly due to reduced intake alongside co-morbidities such as alcoholism, poor mental health or financial vulnerability. In children, similar restrictions may be less obvious but just as common, such as neurobehavioural disorders, a selective or restricted diet and delayed weaning in infancy to solid foods containing vitamin C [3]. While vitamin C deficiency in the modern day may be a rare clinical presentation, it is often overlooked and could lead to unnecessary investigations being carried out, while the diagnosis may have been obvious from the history alone.

## Case presentation

A boy aged three years and eight months presented to acute paediatric services in Gloucester with isolated right knee pain. His previous medical history was unremarkable; he was born at term with no complications and was usually fit and well, achieving his expected neurodevelopmental milestones appropriately.

**Figure 1:**
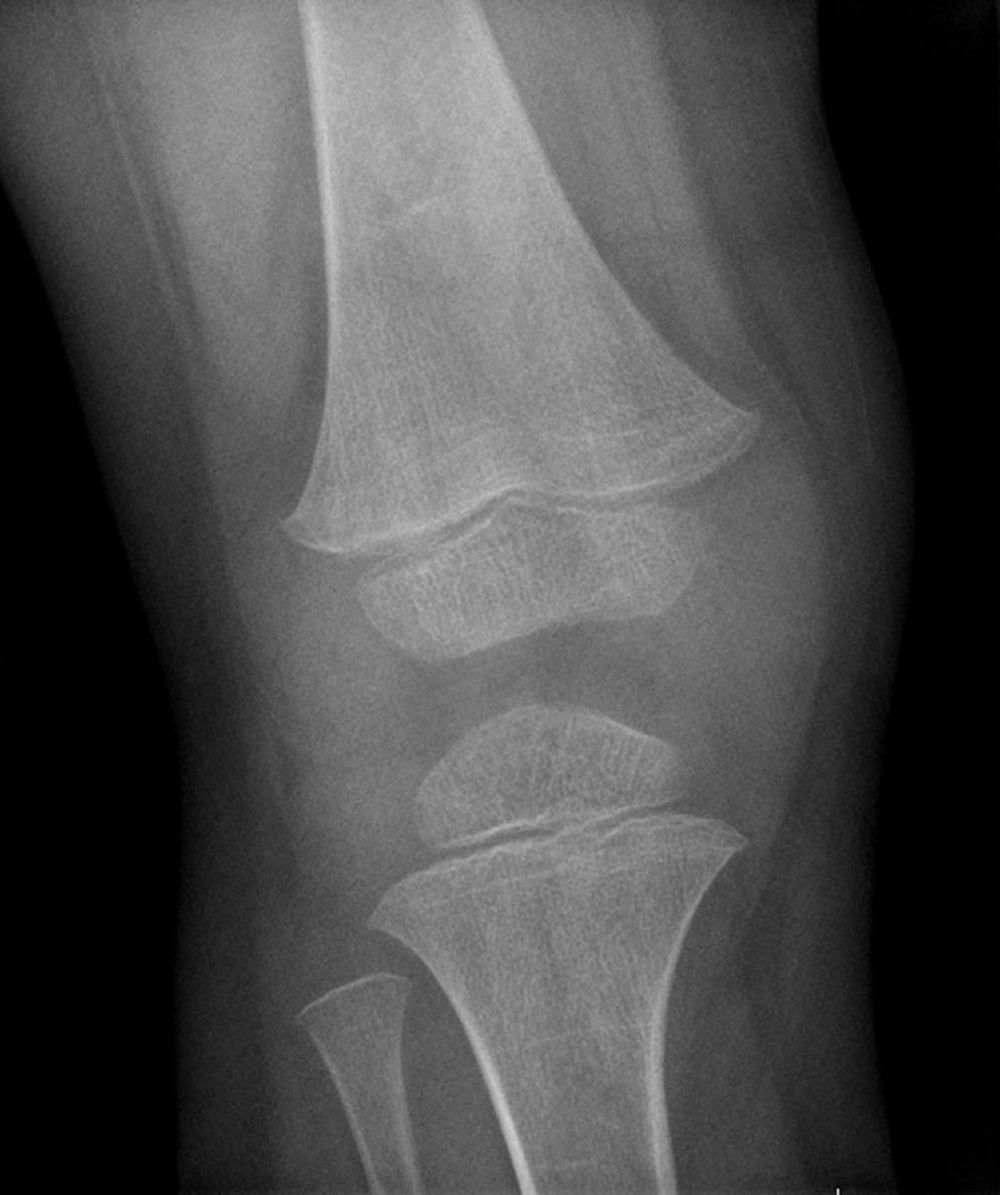
X-ray of the right knee from initial presentation to hospital

**Figure 2:**
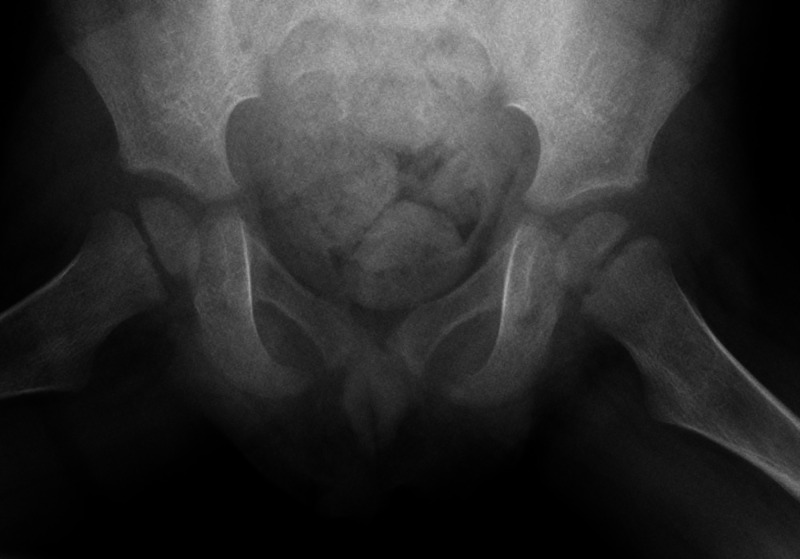
X-ray of the pelvis (frog leg lateral) from initial presentation to hospital

On his first presentation, he was assessed by the orthopaedic team and it transpired that he had been having difficulty weight-bearing for three weeks, with a notable antalgic gait. There was no history of febrile illness but he was recovering from a recent coryzal and flu-like illness. On examination, it was unclear where the pain was coming from, but the right knee was unremarkable with no erythema, effusion or bony tenderness and a full range of movement. Bloods tests, including full blood count, renal and liver function, were normal except for a slightly low albumin (35 g/L) and alkaline phosphatase (79 units/L), and a slightly raised C-reactive protein at 7 mg/L. Plain film x-rays of both right knee and pelvis (Figures 1, 2) were reported as normal, and he was discharged home following a diagnosis of transient synovitis (irritable hip), with planned orthopaedic follow-up in the near future.

He presented for a second time to paediatrics a month later via rapid-access referral from primary care with worsening left hip and knee pain which had started to wake him at night. There was an understandable concern of possible malignancy, juvenile idiopathic or septic arthritis. He continued to have a reassuring examination with a full range of movement in all joints, no provokable bone pain and there was still no history of fever or lethargy. Inflammatory markers on blood testing remained grossly unchanged alongside normal clotting, bone profile and electrolyte studies, although he did have a mild thrombophilia. An ultrasound scan of both the left hip and knee showed neither effusion nor synovial thickening, and he was discharged home once his pain was under control with ibuprofen and with the orthopaedic follow-up expedited.

A week later, he presented for a third time via the hospital emergency department with all-over joint pain, an unusual rash and new bruising. As he was clerked in by a junior doctor, a background of fussy eating was elicited from the history and he was noted to possibly be small for age (height and weight plotted just below the 25th centile). The bruises had appeared without any preceding history of trauma, and he had also been suffering with bleeding gums. At this point he was unwilling to get out of bed and seemed to be in significant amounts of pain which did not localise to any particular joint or muscle group. On examination, all of his joints had a full range of movement passively with no worsening of the pain. He had a polymorphic, partially non-blanching maculopapular rash across his trunk and lower limbs which convalesced with unusual-appearing bruises, along with evidence of significant dental decay and gum hypertrophy. The initial list of differential diagnoses included atypical Henoch-Schonlein purpura, systemic juvenile idiopathic arthritis, a connective tissue disease, clotting disorder and possible vitamin C deficiency.

**Figure 3:**
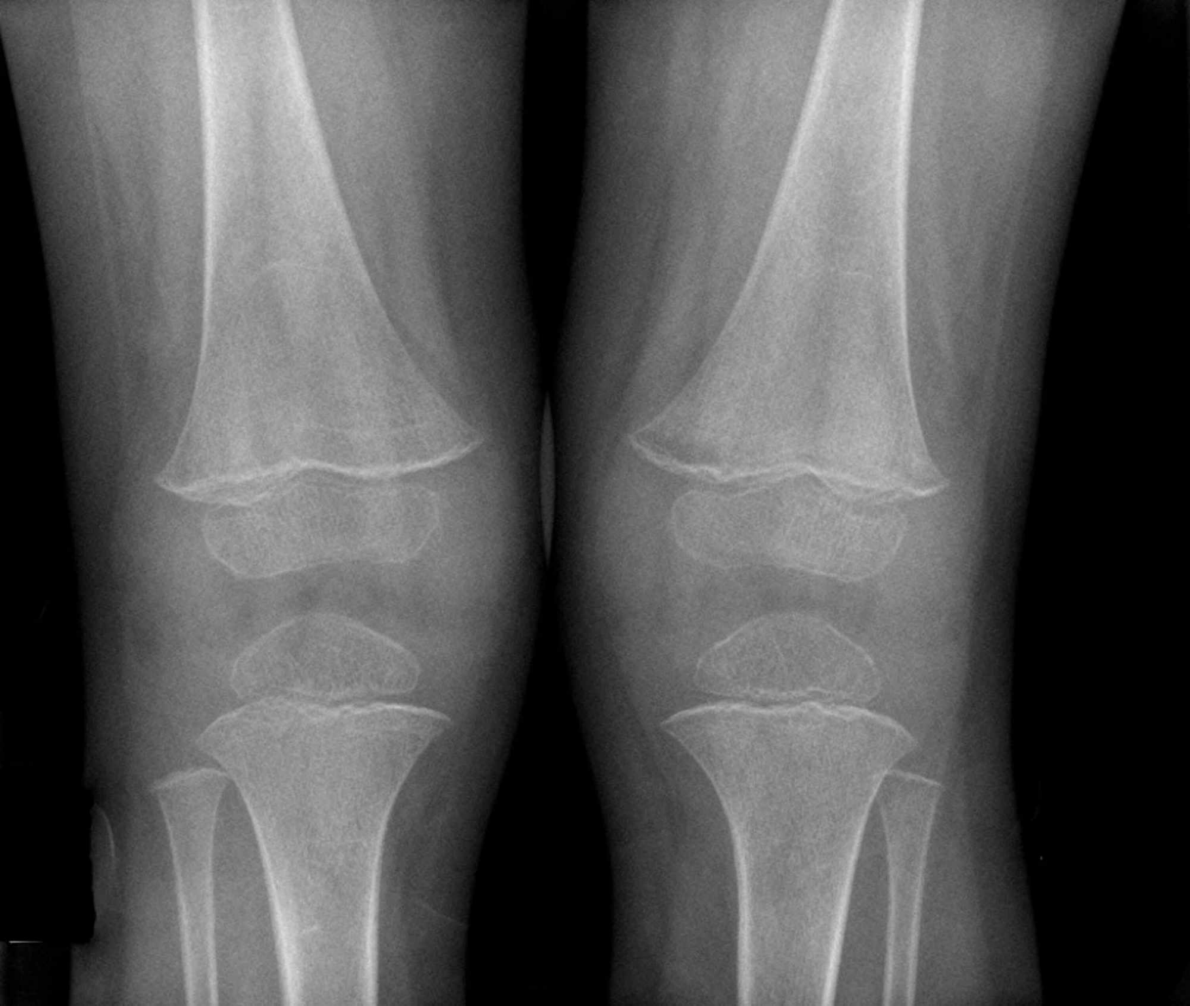
X-ray of both the knees from third presentation to hospital

**Figure 4:**
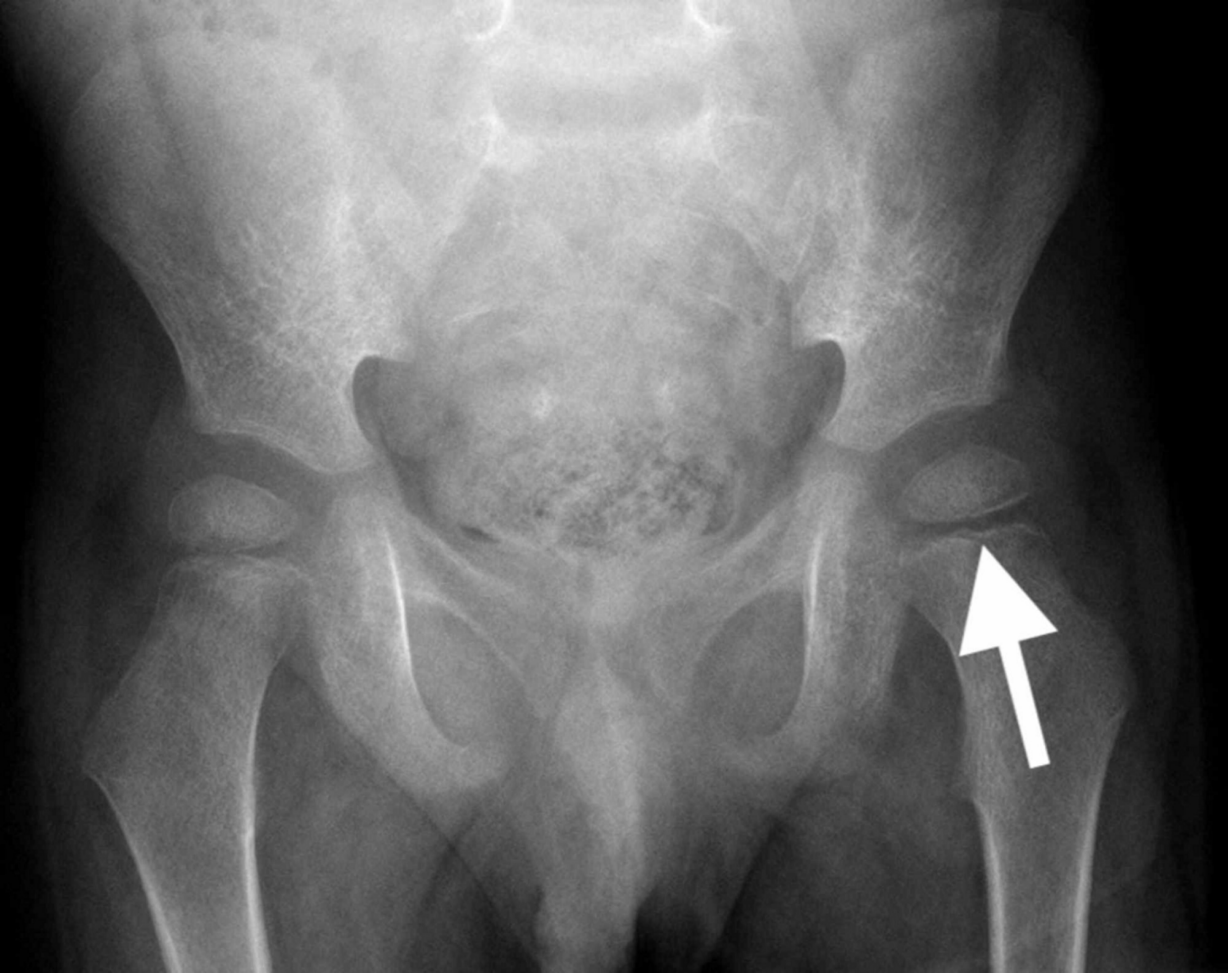
X-ray of the pelvis (anteroposterior view) from third presentation to hospital (arrow pointing to a subcortical osteoporotic ring within the femoral capital epiphysis which is more subtle in this view)

**Figure 5:**
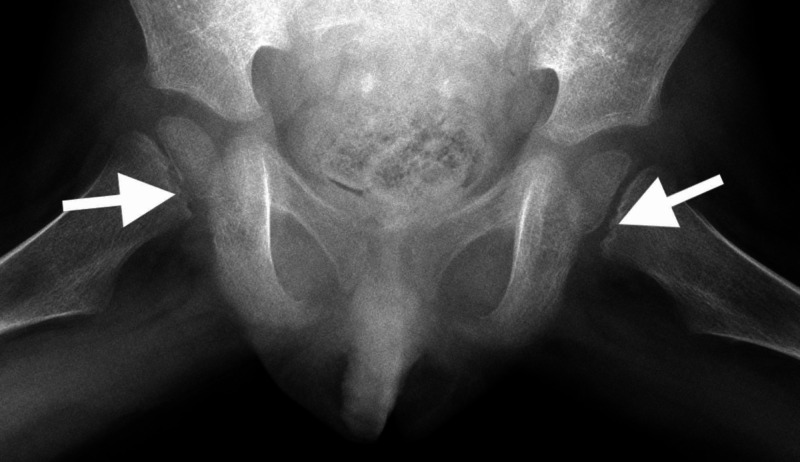
X-ray of the pelvis (frog leg lateral) from third presentation to hospital (arrows pointing to subcortical osteoporotic rings within both femoral capital epiphyses)

Repeat baseline blood tests (full blood count, renal and liver function) remained normal with static inflammatory markers. Additional tests, including thyroid function, ferritin, creatine kinase, vitamins A, D and E, and clotting studies, were also normal. A full autoantibody screen was negative, alongside normal complement levels, but the erythrocyte sedimentation rate (ESR) was slightly raised at 19 mm/hr. Although it took two weeks for the vitamin C assay to be processed, the serum level was significantly low at <2.8 µmol/L (normal range of 26.1-84.6 µmol/L), supporting the diagnosis of vitamin C deficiency, also known as scurvy. X-rays of both the knees (Figure 3) remained unremarkable, but repeat x-ray of the pelvis now showed evidence of subcortical osteoporotic rings within the femoral capital epiphysis (Figures 4, 5), which is consistent with the diagnosis.

With this in mind, a more focused dietetic review was sought. This found that there had been behavioural issues around food from the point of weaning onto solids, and that for several years his nutritional intake had been severely limited to a selective diet of non-fortified cereals, biscuits, chocolate and peanuts, with refusal to take anything else. The family was introduced to behaviour modification strategies, including reward systems to broaden his diet, and he was prescribed oral ascorbic acid (vitamin C) 250 mg once daily alongside a tablet multivitamin.

On review six weeks after diagnosis and starting treatment, his pain and skin manifestations had completely gone and his mobility is slowly returning to normal, more than likely delayed due to the prolonged time that he spent not fully weight-bearing and sedentary.

## Discussion

While early findings of vitamin C deficiency are non-specific, the later musculoskeletal signs and symptoms are common and include myalgia, haemarthroses and haematomas [4]. The bleeding tendency is caused by poor collagen formation within blood vessel walls, and it is likely that subperiosteal haematomas were the elusive cause of the pain our patient experienced. Blood tests in vitamin C deficiency are often variable with no one diagnostic picture other than the low assay level which can take some time to come back. The most commonly consistent finding is an anaemia which may often be haemolytic in nature. Radiological changes have been found in some cases, including cortical thinning, osteopaenia and osteonecrosis. While our patient only manifested symptoms associated with vitamin C deficiency and these completely resolved with its replacement, it is worth considering how his limited diet may have affected other micronutrient availability. Proactively testing for other deficiencies while treating the known deficiency is just as important for longer-term health benefits and ensures we do not miss a contributing cause. Vitamin C deficiency is for most of the population a preventable disease and can be quickly and effectively treated once diagnosed, but needs to be considered within the broad list of differentials for an atypical presentation such as this one [5].

## Conclusions

On reflection, it is clear that a holistic approach to most atypical clinical scenarios will usually extract relevant information that can provide some clarity and lead to a diagnosis. The mention of fussy eating alongside musculoskeletal pain and cutaneous manifestations was enough for the team to pose the question of underlying dietary issues and ultimately vitamin C deficiency, which lead to more appropriate lines of investigation and ultimately the correct diagnosis and effective treatment without further testing being done. Clinicians should be remain vigilant to the role of a child’s diet within the context of their health and especially when their symptoms could be explained by nutritional deficiency, such as a limp in vitamin C deficiency or bone and joint pain in rickets (vitamin D deficiency).
